# Comparison between enzyme-linked immunosorbent assay and indirect immunofluorescence for detection of antineutrophil cytoplasmic antibodies

**DOI:** 10.31744/einstein_journal/2020AO5132

**Published:** 2020-01-22

**Authors:** Julia Miranda Menezes, Raissa Rossener, Ana Paula Marques Aguirra da Silva, Silvia Sanches Rodrigues, Cristóvão Luis Pitangueira Mangueira

**Affiliations:** 1 Faculdade Israelita de Ciências da Saúde Albert Einstein Hospital Israelita Albert Einstein São PauloSP Brazil Faculdade Israelita de Ciências da Saúde Albert Einstein, Hospital Israelita Albert Einstein, São Paulo, SP, Brazil.; 2 Hospital Israelita Albert Einstein São PauloSP Brazil Hospital Israelita Albert Einstein, São Paulo, SP, Brazil.

**Keywords:** Autoimmune diseases, Autoantibodies, Antibodies, antineutrophil cytoplasmic, Enzyme-linked immunosorbent assay, Fluorescent antibody technique, indirect

## Abstract

**Objective:**

To evaluate the performance of enzyme-linked immunosorbent assay and indirect immunofluorescence methods for the detection of antineutrophil cytoplasmic antibodies in a routine clinical laboratory setting.

**Methods:**

A total of 227 samples were tested by indirect immunofluorescence and enzyme-linked immunosorbent assay with antigen specificity for antiproteinase 3 and antimyeloperoxidase. The proportions of positive samples were compared by McNemar hypotheses and agreement was described by Cohen’s Kappa coefficient.

**Results:**

The agreement of the tests was 96.5%, and the Kappa coefficient obtained was 0.70 (95%CI: 0.50-0.90; p<0.001). Considering indirect immunofluorescence as the gold standard, the sensitivity of the enzyme-linked immunosorbent assay was 0.62 and the specificity was 0.99, with diagnostic accuracy in 96% of cases. Some samples were negative in enzyme-linked immunosorbent assay and positive in indirect immunofluorescence. This situation occurred in all immunofluorescence patterns, but particularly in atypical patterns. Two samples with antiproteinase 3 positivity were considered negative in indirect immunofluorescence.

**Conclusion:**

The enzyme-linked immunosorbent assay had high specificity but lower sensitivity. The performance of indirect immunofluorescence increases diagnostic sensitivity, while the search for antiproteinase 3 by enzyme-linked immunosorbent assay may also add diagnostic power.

## INTRODUCTION

The detection and characterization of autoantibodies in serum is a critical part of the clinical diagnosis of several autoimmune diseases. However, reference clinical laboratories permanently face the dilemma of choosing among the different methods available, which can be used for screening or confirmation, alone or combined. These commercial tests differ in terms of the nature of the substrate, conditions for the action of antibodies, and mode of detection of markers, which can affect their sensitivity and specificity. For this reason, there is considerable variability in their performance, with conspicuous differences such as, for example, when comparing the enzyme-linked immunosorbent assay (ELISA) and indirect immunofluorescence (IIF), which can interfere in clinical decision-making.^1^

The group of antineutrophil cytoplasmic autoantibodies, known as ANCA, is a relevant serum marker for a group of diseases called systemic vasculites, such as granulomatosis with polyangiitis (GPA), microscopic polyangiitis (MPA) and eosinophilic granulomatosis with polyangiitis (EGPA),^2 , 3^ in addition to having an ancillary role in the differential diagnosis of inflammatory bowel disease.^4^

The classic method used by most laboratories for ANCA testing around the world is IIF with a substrate of ethanol and/or formalin-fixed human neutrophils. This test is commonly used before confirmatory testing for antigen specificities in the same laboratory where the screening was carried out, or in reference laboratories.^5 , 6^ Samples considered positive in microscopy can show three distinct patterns (cytoplasmic – cANCA, perinuclear – pANCA or atypical – aANCA,), depending on the fluorescence characteristics. The cytoplasmic pattern is more frequently caused by antiproteinase 3 (PR-3) antibodies, and is associated with a GPA diagnosis.^7^ The pANCA pattern, on the other hand, may be related with different autoantibodies of distinct specificities, of which the most clinically relevant are antimyeloperoxidase (MPO) antibodies,^8^ and is more frequently associated with a MPA diagnosis.^1^ Other antigens associated with pANCA include cathepsin G, lactoferrin and elastase, for example.^8^ These same autoantibodies, however, could also give rise to a cytoplasmic pattern in some patients.^9^ The pANCA pattern is an artifact of the technique, caused by ethanol fixation, in which the granule membranes burst, and positively charged proteins migrate to the negatively charged nucleus.^8^ The aANCA pattern, in turn, is mixed, and combines cytoplasmic and perinuclear or nuclear staining, with multiple antigenic specificities.^1^

Previous studies have shown that the specificity of IIF for ANCA testing is close to 93%, whereas sensitivity is approximately 67% to 78%, and additional tests are needed to improve this performance.^4^ ELISA can play this role.^4^ In this case, proteins with individual antigen specificities are used − particularly PR-3 and MPO.^1^ The specific determination of anti-PR3 and anti-MPO antibodies is useful in differential diagnosis of systemic vasculites, particularly when differentiating between GPA, MPA and EGPA, which have diverse clinical presentations, prognoses and treatment responses.^10^

The reason for the mismatch between IIF and ELISA results when testing for ANCA is still unknown. One hypothesis is that IIF can detect antibodies against proteins that are still unknown, and these antibodies cannot be detected by ELISA, since it uses specific proteins.^11^

Although the classic method for ANCA testing is IIF followed by confirmatory ELISA, some recent studies have challenged the need for the IIF test,^12^ whereas others recommend the simultaneous use of both methods which, when combined, could result in a sensitivity and specificity of 92% and 99%, respectively.^4^

There is a need to revise the current diagnostic algorithm used for ANCA detection.^1 , 13 , 14^ In this light, this study compared the results of the ELISA and IIF methods for ANCA testing in blood samples collected in a clinical laboratory, which is considered as a reference for this type of testing, in patients at different stages of diagnostic investigation for several suspected conditions.

## OBJECTIVE

To investigate the performance of the enzyme-linked immunosorbent assay and indirect immunofluorescence methods for detection of antineutrophil cytoplasmic antibodies in a reference clinical laboratory for autoimmunity testing, comparing the current algorithm in use with other hierarchization strategies involving indirect immunofluorescence and enzyme-linked immunosorbent assay.

## METHODS

This study used 227 serum samples from patients seen at the clinical laboratory of *Hospital Israelita Albert Einstein* (HIAE), between April and October 2016, for whom ANCA testing had been requested by their attending physicians, within the context of their clinical investigation. Blood samples were obtained with a standard vacuum collection system (Sarstedt, Germany) used in the hospital, and centrifuged for serum separation, as per the routine established for ANCA testing at the laboratory. All serum samples were tested by the two methods: IIF (Euroimmun^®^, Germany) and anti-PR3 and anti-MPO ELISA (Inova, Werfen^®^, USA). The results were entered in a spreadsheet for comparison purposes.

ELISA tests used diagnostic kits with purified human anti-PR3 and anti-MPO antigens, previously bound to polystyrene plates (Inova, Werfen^®^, USA). Controls and pre-diluted patient sera were added to the different wells, allowing any anti-PR3 and anti-MPO antibodies present to separately bind to the immobilized antigens. After the washing step, enzyme-labeled anti-human IgG conjugate was added to each well. A second incubation allowed the enzyme conjugate to bind to any patient antibodies adhered to the wells. After the second wash, to remove any excess conjugate, the remaining enzyme activity was determined by adding a specific chromogenic substrate and measuring the color intensity by spectrometry, to compare the color intensity of patient wells and control wells. In this case, samples were considered positive if they reacted to anti-PR3 or anti-MPO. The cutoff used for both tests was 20 units. Positive samples were further classified into weak positives (21 to 30 units) and moderate to strong positives (over 30 units).

IIF tests used diagnostic kits with ethanol-fixed human neutrophils (Euroimmun^®^, Germany). In this test, patient serum was added to slides with a pre-fixed substrate. In a second step, fluorescein-labeled antibodies (conjugate) against patient antibodies were added. The slides were read in a microscope by two independent observers and classified into “non reagent” (no fluorescence) or “reagent” (if fluorescence was present). “Reagent” samples were classified into three possible patterns of fluorescence: cANCA, pANCA or aANCA. The cANCA pattern is detected by fluorescence in the cytoplasm of segmented neutrophils; in pANCA, fluorescence is seen around the nuclei of neutrophils; aANCA, in turn, shows different patterns, or a combination of the previous patterns.

In the statistical analysis, the proportions of positive samples observed in each of the tests were compared by Mc Nemar’s test (for dependent data). The rate of agreement between the tests was described by the proportion of agreement and Cohen’s kappa coefficient, using a 95% confidence interval (95%CI) and a p-value for hypothesis testing. Statistical analyses were conducted with the aid of computing package R (R Core Team, 2017), version 3.4.1, assuming a significance level of 5%.

This study was approved by the Institutional Review Board (IRB) under final opinion number 2.939.366 and CAAE: 70390417.5.0000.0071. The waiver of informed consent form was approved under opinion number 2.274.307.

## RESULTS

Of the 227 samples tested, 12 (5.29%) were positive in ELISA and 16 (7.05%) on IIF. This difference was not significant in the McNemar’s hypothesis test (p=0.289). Only 10 (4.4%) samples were positive in both methods ( Table 1 ).

**Table 1:** General description of results for antineutrophil cytoplasmic antibodies

Results	n (%)	Median (minimum - maximum)
IIF		
Negative	211 (93.0)	
Positive	16 (7.0)	
Pattern of positive tests		
aANCA	3 (1.3)	
cANCA	8 (3.5)	
pANCA	5 (2.2)	
Myeloperoxidase (unit)		2.34 (0.97-46.20)
Categories of positive tests		
Up to 20	222 (97.8)	
21-30	3 (1.3)	
>30	2 (0.9)	
Proteinase 3 (unit)		2.40 (0.69-116.00)
Categories of positive tests		
Up to 20	218 (96.0)	
21-30	2 (0.9)	
>30	7 (3.1)	
ELISA		
Negative	215 (94.7)	
Positive	12 (5.3)	
Categories of positive tests		
MPO and PR3 up to 20	215 (94.7)	
MPO or PR3 21-30	3 (1.3)	
MPO or PR3 >30	9 (4.0)	

IIF: indirect immunofluorescence; aANCA: atypical pattern of antineutrophil cytoplasmic antibodies; cANCA: cytoplasmic pattern of antineutrophil cytoplasmic antibodies; pANCA: perinuclear pattern of antineutrophil cytoplasmic antibodies; ELISA: enzyme-linked immunosorbent assay; MPO: myeloperoxidase; PR3: proteinase 3.

**Figure 1:**
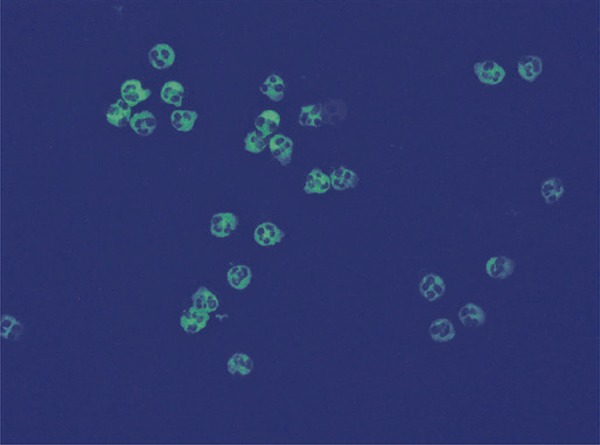
Cytoplasmic pattern observed under indirect immunofluorescence in ethanol-fixed human neutrophils. Apple-green fluorescence can be seen throughout the cytoplasm of segmented neutrophils, with negative nuclei

**Figure 2:**
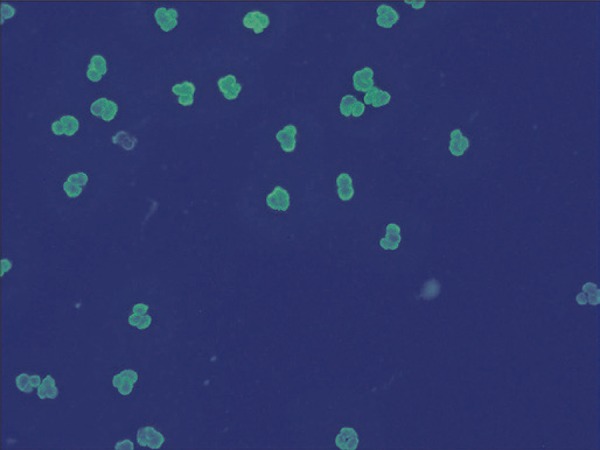
Perinuclear pattern observed under indirect immunofluorescence in ethanol-fixed human neutrophils. Apple-green fluorescence can be seen around and over the nuclei of segmented neutrophils, with the rest of the cytoplasm being negative

**Figure 3:**
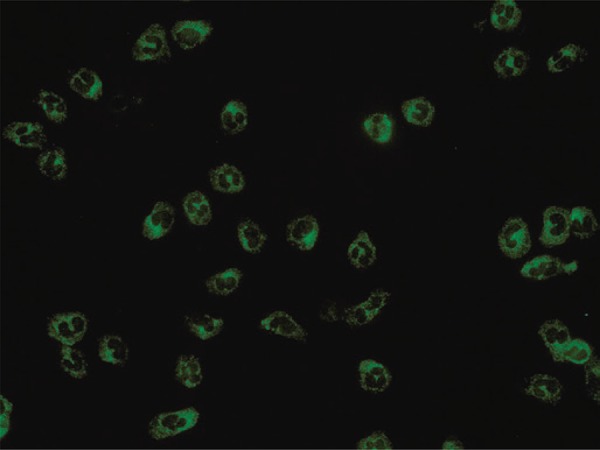
Atypical pattern observed under indirect immunofluorescence in ethanol-fixed human neutrophils. Apple-green fluorescence can be seen, with features that do not define it as one of the two classic fluorescence patterns (cytoplasmic or perinuclear)

Of the 16 positive cases on IIF, 8 were classified as cANCA ( Figure 1 ), of which 6 were positive for anti-PR3 and 2 were negative in ELISA; 5 cases were classified as pANCA ( Figure 2 ), of which 3 were positive for anti-MPO, 1 positive for anti-PR3 and 1 negative in both ELISA tests; three were classified as aANCA ( Figure 3 ), and also negative in ELISA.

Additionally, two negative cases under IIF were positive in the ELISA anti-PR3 test, one with a value between 21 and 30, and one over 30 units ( Table 2 ).

**Table 2:** Results of the enzyme-linked immunosorbent assay for antineutrophil cytoplasmic antibodies

Enzyme-linked immunosorbent assay	Indirect immunofluorescence				

	Negative	Positive	aANCA	cANCA	pANCA
Negative	209 (99.1)	6 (37.5)	3 (100.0)	2 (25.0)	1 (20.0)
Positive	2 (0.9)	10 (62.5)	0 (0.0)	6 (75.0)	4 (80.0)
Categories of positive tests					
MPO and PR3 up to 20	209 (99.1)	6 (37.5)	3 (100.0)	2 (25.0)	1 (20.0)
MPO or PR3 21 - 30	1 (0.5)	2 (12.5)	0 (0.0)	1 (12.5)	1 (20.0)
MPO or PR3 > 30	1 (0.5)	8 (50.0)	0 (0.0)	5 (62.5)	3 (60.0)
Myeloperoxidase (unit)					
Up to 20	211 (100.0)	11 (68.8)	3 (100.0)	6 (75.0)	2 (40.0)
21-30	0 (0.0)	3 (18.8)	0 (0.0)	2 (25.0)	1 (20.0)
>30	0 (0.0)	2 (12.5)	0 (0.0)	0 (0.0)	2 (40.0)
Serine protease 3 (unit)					
Up to 20	209 (99.1)	9 (56.2)	3 (100.0)	2 (25.0)	4 (80.0)
21-30	1 (0.5)	1 (6.2)	0 (0.0)	1 (12.5)	0 (0.0)
>30	1 (0.5)	6 (37.5)	0 (0.0)	5 (62.5)	1 (20.0)

Results expressed as n (%). aANCA: atypical pattern of antineutrophil cytoplasmic antibodies; cANCA: cytoplasmic pattern of antineutrophil cytoplasmic antibodies; pANCA: perinuclear pattern of antineutrophil cytoplasmic antibodies; MPO: myeloperoxidase; PR3: proteinase 3.

The two methods were in agreement for 10 positive samples and 209 negative samples, leading to an agreement of 96.5% and a Kappa coefficient of 0.70 (95%CI: 0.50-0.90; p<0.001). This value indicates substantial, however not perfect agreement.

Table 3 shows the diagnostic measurements of the ELISA test for ANCA, with IIF as the gold standard. Its sensitivity was 0.62 and specificity, 0.99, which makes it a more specific than sensitive test, with diagnostic accuracy in 96% of cases.

**Table 3:** Diagnostic measurements of the enzyme-linked immunosorbent assay for antineutrophil cytoplasmic antibodies, according to indirect immunofluorescence

Diagnostic measurements	Estimate
Sensitivity	0.62 (0.35-0.85)
Specificity	0.99 (0.97-1.00)
Positive predictive value	0.83 (0.52-0.98)
Negative predictive value	0.97 (0.94-0.99)
Diagnostic accuracy	0.96 (0.93-0.98)

The measurements were assessed based on contingency tables.

## DISCUSSION

Samples negative in ELISA and positive in IIF were found for all patterns of fluorescence (cANCA, pANCA and aANCA), however more frequently for aANCA patterns. This finding differs from the findings of Damoiseaux et al., who suggested there was no advantage in using IIF for screening or in association with ELISA, when it came to finding ANCA patterns in patients with suspected vasculites.^12^

Harris et al., demonstrated that, in cases of active, biopsy-confirmed, systemic necrotizing vasculitis, the ELISA method had similar sensitivity (85% *versus* 88%; p=0.056), however greater specificity (97% *versus* 90%; p=0.0006) and positive predictive value (73% *versus* 50%; p=0.0013) than IIF for ANCA detection. The authors also showed that the combination of immunofluorescence and ELISA led to a lower positive predictive value when compared with ELISA alone.^15^ Our results, however, suggest that the use of IIF in all cases referred for ANCA detection would increase the sensitivity of testing, since it would classify as positive patients who, without IIF, would be considered negative.

On the other hand, the occurrence of two negative samples in IIF with positive results for anti-PR3, an autoantibody of high clinical relevance for GPA diagnosis, suggests that anti-PR3 testing with ELISA, previously or simultaneously to the IIF test, can improve diagnostic power, as supported by previous studies.^4 , 6^

These data show the need for revision of the current algorithm, supporting the findings of previous studies.^13 , 14^

Our study, however, has limitations, such as the failure to evaluate the correlation between laboratory results and the clinical diagnosis of the study subjects. We plan to assess this correlation in future studies. Also, IIF was investigated as a gold standard, and it was not possible to estimate the sensitivity and specificity of this method alone.

In our case series, the combined, simultaneous use of ELISA and IIF was deemed the most appropriate diagnostic strategy, conferring the highest sensitivity to ANCA testing, and including as positive all patients with autoantibodies of antigen specificities relevant to the differential diagnosis of systemic vasculites, particularly those positive for anti-PR3.

## CONCLUSION

Results show that the antimyeloperoxidase and antiproteinase 3 ELISA tests used together had high specificity, however with inferior sensitivity.

It is possible to say that indirect immunofluorescence increased the sensitivity of ANCA testing when compared with ELISA alone and, at the same time, the antiproteinase 3 ELISA test improved the sensitivity for samples with this antigen specificity. Based on these results, it is possible to suggest that the combined, simultaneous use of indirect immunofluorescence and ELISA is the most appropriate diagnostic strategy for the population studied.

## References

[1] 1. Csernok E, Moosig F. Current and emerging techniques for ANCA detection in vasculitis. Nat Rev Rheumatol. 2014;10(8):494-501. Review. 10.1038/nrrheum.2014.78 24890776

[2] 2. McKinney EF, Willcocks LC, Broecker V, Smith KG. The immunopathology of ANCA-associated vasculitis. Semin Immunopathol. 2014;36(4):461-78. Review. 10.1007/s00281-014-0436-6 PMC4118034 25056155

[3] 3. Lamprecht P, Kerstein A, Klapa S, Schinke S, Karsten CM, Yu X, et al. Pathogenetic and Clinical Aspects of Anti-Neutrophil Cytoplasmic Autoantibody-Associated Vasculitides. Front Immunol. 2018;9:680. Review. 10.3389/fimmu.2018.00680 PMC5900791 29686675

[4] 4. Suwanchote S, Rachayon M, Rodsaward P, Wongpiyabovorn J, Deekajorndech T, Wright HL, et al. Anti-neutrophil cytoplasmic antibodies and their clinical significance. Clin Rheumatol. 2018;37(4):875-84. Review. 10.1007/s10067-018-4062-x 29525845

[5] 5. Savige J, Davies D, Falk RJ, Jennette JC, Wiik A. Antineutrophil cytoplasmic antibodies and associated diseases: a review of the clinical and laboratory features. Kidney Int. 2000;57(3):846-62. Review. 10.1046/j.1523-1755.2000.057003846.x 10720938

[6] 6. Bossuyt X, Cohen Tervaert JW, Arimura Y, Blockmans D, Flores-Suárez LF, Guillevin L, et al. Position paper: Revised 2017 international consensus on testing of ANCAs in granulomatosis with polyangiitis and microscopic polyangiitis. Nat Rev Rheumatol. 2017;13(11):683-92. Review. 10.1038/nrrheum.2017.140 28905856

[7] 7. Niles JL, McCluskey RT, Ahmad MF, Arnaout MA. Wegener’s granulomatosis autoantigen is a novel neutrophil serine proteinase. Blood. 1989;74(6):1888-93. 2679910

[8] 8. Falk RJ, Jennette JC. Anti-neutrophil cytoplasmic autoantibodies wilh specificity for myeloperoxidase in patients with systemic vasculitis and idiopathic nccrotizing and crescentic gtomerulonephritis. N Engl J Med. 1988;318(25):1651-7. 10.1056/NEJM198806233182504 2453802

[9] 9. Segelmark M. Baslund B, Wieslander J. Some patients with anti-myeioperoxidase autoantibodies have a c-ANCA pattern. Clin Exp Immunol. 1994;96(3):458-65. 10.1111/j.1365-2249.1994.tb06051.x PMC1534554 7516271

[10] 10. Cohen Tervaert JW, Damoiseaux J. Antineutrophil cytoplasmic autoantibodies: how are they detected and what is their use for diagnosis, classification and follow-up? Clin Rev Allergy Immunol. 2012;43(3):211-9. Review. 10.1007/s12016-012-8320-4 22669754

[11] 11. Baslund B, Segelmark M, Wiik A, Szpirt W, Petersen J, Wieslander J. Screening for anti-neutrophil cytoplasmic antibodies (ANCA): is indirect immunofluorescence the method of choice? Clin Exp Immunol. 1995;9(3):486-92. 10.1111/j.1365-2249.1995.tb05577.x PMC1534202 7882573

[12] 12. Damoiseaux J, Csernok E, Rasmussen N, Moosig F, van Paassen P, Baslund B, et al. Detection of antineutrophil cytoplasmic antibodies (ANCAs): a multicentre European Vasculitis Study Group (EUVAS) evaluation of the value of indirect immunofluorescence (IIF) versus antigen-specific immunoassays. Ann Rheum Dis. 2017;76(4):647-53. 10.1136/annrheumdis-2016-209507 27481830

[13] 13. Bueno C, Bonfá ED, Radu AS, Cossermelli W. [Comparison between immunofluorescence techniques and ELISA, using whole neutrophil extract and primary granules, for the detection of antineutrophil cytoplasma antibodies (ANCA).] Rev Hosp Clin Fac Med Sao Paulo. 1995;50(2):101-6. Portuguese. 7569596

[14] 14. Godbole MS, Valenzuela R, Deodhar SD, Calabrese L, Tubbs RR. Comparative study of ELISA and indirect immunofluorescence for the detection of anti-neutrophil cytoplasmic antibodies: evaluation of the SCIMEDX/EURO Diagnostica ELISA assay in a clinical setting. Am J Clin Pathol. 1995;104(6): 667-72. 10.1093/ajcp/104.6.667 8526211

[15] 15. Harris A, Chang G, Vadas M, Gillis D. ELISA is the superior method for detecting antineutrophil cytoplasmic antibodies in the diagnosis of systemic necrotising vasculitis. J Clin Pathol. 1999;52(9):670-6. 10.1136/jcp.52.9.670 PMC501542 10655988

