# Supplementary material for: Comparison between enzyme-linked immunosorbent assay and indirect immunofluorescence for detection of antineutrophil cytoplasmic antibodies
Source: Einstein (Sao Paulo). 2020 Jan 22;18:eAO5132. doi: 10.31744/einstein_journal/2020AO5132 (PMC6980292; doi:10.31744/einstein_journal/2020AO5132)
Supplement: Translation

# Translation: Comparação entre os métodos de ensaio imunoenzimático e imunoflurescência indireta para a pesquisa de anticorpos anticitoplasma de neutrófilos

**Authors:** Julia Miranda Menezes, Raissa Rossener, Ana Paula Marques Aguirra da Silva, Silvia Sanches Rodrigues, Cristóvão Luis Pitangueira Mangueira

**Affiliations:** 1 Brasil Faculdade Israelita de Ciências da Saúde Albert Einstein, Hospital Israelita Albert Einstein, São Paulo, SP, Brasil.; 2 Brasil Hospital Israelita Albert Einstein, São Paulo, SP, Brasil.

**Keywords:** Doenças autoimunes, Autoanticorpos, Anticorpos, anticitoplasma de neutrófilos, Ensaio de imunoadsorção enzimática, Técnica indireta de fluorescência para anticorpo

## Abstract

**Objetivo:**

Avaliar o desempenho das metodologias de ensaio imunoenzimático e imunofluorescência indireta para a detecção de anticorpos anticitoplasma de neutrófilos em um contexto de laboratório clínico de rotina.

**Métodos:**

Foram testadas 227 amostras pelas metodologias de imunofluorescência indireta e ensaio imunoenzimático com especificidades para anticorpos antiproteinase-3 e antimieloperoxidase. As proporções de amostras positivas foram comparadas por hipóteses de McNemar, e a concordância foi descrita pelo coeficiente Kappa de Cohen.

**Resultados:**

A concordância dos testes foi 96,5%, e o coeficiente Kappa obtido foi 0,70 (IC95%: 0,50-0,90; p<0,001). Utilizando a imunofluorescência indireta como padrão-ouro, a sensibilidade do ensaio imunoenzimático foi de 0,62 e a especificidade, 0,99, com acurácia diagnóstica em 96% dos casos. Algumas amostras apresentaram resultados negativos por ensaio imunoenzimático e positivos por imunofluorescência. Isso ocorreu em amostras com vários padrões de fluorescência, mas particularmente nos casos com padrões atípicos. Duas amostras com positividade antiproteinase 3 foram consideradas negativas por imunofluorescência.

**Conclusão:**

Os métodos de ensaio imunoenzimático tiveram alta especificidade, mas sensibilidade inferior. A realização da imunofluorescência indireta aumenta a sensibilidade diagnóstica, ao mesmo tempo que a pesquisa de antiproteinase 3 por ensaio imunoenzimático também pode agregar poder diagnóstico.

## INTRODUÇÃO

A detecção e a caracterização sorológica de autoanticorpos é parte fundamental do diagnóstico clínico de várias doenças autoimunes. Entretanto, os laboratórios clínicos de referência convivem permanentemente com o dilema de escolher entre os diferentes métodos disponíveis, que podem ser utilizados como triagem ou confirmação dos achados, isoladamente ou em conjunto. Esses testes comerciais diferem em relação à natureza do substrato, às condições para ação dos anticorpos e ao modo de detecção dos marcadores, o que pode afetar a sensibilidade e a especificidade de cada um. Em virtude disso, há considerável variabilidade em seus desempenhos, com diferenças evidentes, por exemplo, entre métodos de ensaio imunoenzimático (ELISA) e de imunofluorescência indireta (IFI), que podem interferir na elaboração de decisões clínicas.^1^

O grupo de autoanticorpos anticitoplasma de neutrófilos, conhecido como ANCA, é um relevante marcador sorológico de um grupo de enferimidades conhecidas como vasculites sistêmicas, como a granulomatose com poliangeíte (GPA), a poliangeíte microscópica (MPA) e a granulomatose eosinofílica com poliangeíte (EGPA),^2 , 3^ além de desempenhar papel acessório no diagnóstico diferencial de doenças inflamatórias intestinais.^4^

O método clássico utilizado pela maioria dos laboratórios para a pesquisa de ANCA em todo o mundo é a IFI com substrato de neutrófilos humanos fixados em etanol e/ou formalina. Esse teste é comumente utilizado previamente à realização de métodos confirmatórios para especificidade antigênicas no próprio laboratório que realizou a triagem ou em laboratórios de referência.^5 , 6^ As amostras consideradas positivas à microscopia podem se apresentar em três padrões distintos (citoplasmático – cANCA, perinuclear – pANCA ou atípico – aANCA,), a depender das características de fluorescência. O padrão citoplasmático é mais frequentemente causado por anticorpos dirigidos contra a proteinase 3 (PR-3), e associado ao diagnóstico de GPA.^7^ Por outro lado, o pANCA pode estar associado a vários autoanticorpos de especificidades distintas, dentre os quais os mais clinicamente relevantes são os anticorpos contra a mieloperoxidase (MPO),^8^ associados mais frequentemente ao diagnóstico de MPA.^1^ Outros antígenos associados ao pANCA incluem a catepsina G, a lactoferrina e a elastase, por exemplo.^8^ Esses mesmos autoanticorpos, no entanto, também podem dar origem a um padrão citoplasmático em alguns pacientes.^9^ O padrão pANCA é um artefato de técnica causado pela fixação com etanol, em que as membranas dos grânulos se rompem, fazendo com que as proteínas carregadas positivamente migrem para o núcleo carregado negativamente.^8^ O padrão aANCA, por sua vez, é misto e combina colorações citoplasmáticas e perinucleares ou nucleares, apresentando múltiplas especificidades antigênicas.^1^

Estudos anteriores demonstraram que a especificidade da IFI para a detecção de ANCA é próxima a 93%, e a sensibilidade está em torno de 67% a 78%, sendo necessários testes adicionais para melhorar este desempenho.^4^ O ELISA pode realizar esse papel.^4^ Nesse caso, são utilizadas proteínas com especificidades antigênicas individuais − em particular a PR-3 e a MPO.^1^ A determinação específica de anticorpos anti-PR3 e anti-MPO é útil no diagnóstico diferencial das vasculites sistêmicas, particularmente na diferenciação clínica entre GPA, MPA e EGPA, condições com diversas manifestações clínicas, prognóstico e resposta à terapia.^10^

Ainda não se sabe a razão da discrepância entre os resultados obtidos por IFI e ELISA na pesquisa de ANCA. Uma hipótese é que na IFI podem ser detectados anticorpos contra proteínas ainda desconhecidas, os quais não podem ser detectados por ELISA, que utiliza proteínas específicas.^11^

Apesar do método clássico de pesquisa de ANCA ser a IFI seguida por confirmação com ELISA, alguns estudos recentes questionaram a necessidade da realização da IFI,^12^ enquanto outros sugeriram a utilização simultânea dos dois métodos, mostrando que a combinação dos dois testes resultaria em sensibilidade e especificidade de 92% e 99%, respectivamente.^4^

Torna-se relevante rever o algoritmo diagnóstico para a detecção de ANCA adotado atualmente.^1 , 13 , 14^ Diante desse cenário, o presente estudo comparou os resultados dos métodos de ELISA e IFI para pesquisa de ANCA em amostras de sangue coletadas por um laboratório clínico reconhecido como referência para a realização destes testes de pacientes em diferentes estágios de investigação diagnóstica e com suspeitas clínicas variadas.

## OBJETIVO

Avaliar o desempenho das metodologias de ensaio imunoenzimático e imunofluorescência indireta para a detecção de anticorpos anticitoplasma de neutrófilos em um laboratório clínico de referência para exames de autoimunidade, comparando o algoritmo em uso com outras estratégias de hierarquização dos testes de imunofluorescência indireta e ensaio imunoenzimático.

## MÉTODOS

Foram utilizadas 227 amostras de soro colhidas de pacientes atendidos pelo laboratório clínico do Hospital Israelita Albert Einstein (HIAE) entre abril e outubro de 2016, para os quais a pesquisa de ANCA tinha sido solicitada pelos médicos destes pacientes, no contexto de sua investigação clínica. Amostras de sangue foram obtidas por sistema de coleta a vácuo (Sarstedt, Alemanha) padronizado pelo laboratório do hospital e centrifugadas para obtenção do soro, conforme rotina estabelecida para a realização da pesquisa de ANCA pelo laboratório. Todas as amostras de soro foram testadas pelas duas metodologias: IFI (Euroimmun^®^, Alemanha) e ELISA anti-PR3 e anti-MPO (Inova, Werfen^®^, EUA). Os resultados foram inseridos em planilha para a realização de estudos comparativos.

Para a realização dos ensaios de ELISA, foram utilizados *kits* diagnósticos com antígenos humanos anti-PR3 e anti-MPO purificados e previamente ligados a poços de placas de poliestireno (Inova, Werfen^®^, EUA). Controles e soros de pacientes pré-diluídos foram adicionados aos diferentes poços, permitindo que quaisquer anticorpos PR-3 e MPO presentes, separadamente, se ligassem aos antígenos imobilizados. Após etapa de lavagem, adicionou-se conjugado IgG anti-humano marcado com enzima a cada poço. Uma segunda incubação permitiu ao conjugado enzimático ligar-se a quaisquer anticorpos de pacientes aderidos aos poços. Depois da segunda lavagem, para retirar o excesso de conjugado, o restante da atividade das enzimas foi medido, adicionando um substrato cromogênico específico e medindo-se a intensidade da cor que se desenvolve por espectrometria, comparando a intensidade da cor desenvolvida nos poços do paciente com a cor dos poços de controle. Nesse caso, as amostras foram consideradas positivas se reagentes para anti-PR3 ou para anti-MPO. O valor de corte utilizado para ambos os ensaios foi de 20 unidades. Amostras positivas foram subclassificadas em positivas fracas (de 21 a 30 unidades) e positivas moderadas a fortes (acima de 30 unidades).

Para a realização da IFI, utilizaram-se *kits* diagnóstico com neutrófilos humanos fixados com etanol (Euroimmun^®^, Alemanha). Neste ensaio, o soro dos pacientes foi adicionado à lâmina com substrato pré-fixado. Em uma segunda etapa, anticorpos marcados com fluoresceína (conjugados) e direcionados contra os anticorpos do paciente foram adicionados. As lâminas foram lidas ao microscópio por dois observadores independentes e classificadas em “não reagentes” (quando não se observou fluorescência) ou “reagentes” (quando se observou fluorescência). As amostras “reagentes” foram classificadas em três padrões possíveis de fluorescência: cANCA, pANCA ou aANCA. O padrão cANCA é detectado pela fluorescência no citoplasma dos neutrófilos segmentados; o pANCA apresenta fluorescência em torno do núcleo dos neutrófilos; já o aANCA revela diferentes padrões ou uma combinação dos padrões anteriores.

Na análise estatística, foram comparadas as proporções de amostras positivas observadas em cada um dos testes, por meio de testes de hipóteses de McNemar (para dados dependentes). A concordância entre os testes foi descrita pela proporção de concordância e pelo coeficiente de concordância Kappa de Cohen, acompanhado de intervalo de confiança de 95% (IC95%) e do valor de p para o teste de hipóteses. As análises estatísticas foram conduzidas com o auxílio do pacote computacional R (R Core Team, 2017), versão 3.4.1, e adotou-se nível de significância de 5%.

Este estudo foi aprovado pelo Comitê de Ética em Pesquisa (CEP), com parecer final número 2.939.366 e protocolo CAAE: 70390417.5.0000.0071. Foi aceita a isenção do Termo de Consentimento Livre e Esclarecido (TCLE), parecer número 2.274.307.

## RESULTADOS

Das 227 amostras testadas, 12 (5,29%) foram positivas por ELISA e 16 (7,05%) por IFI. Essa diferença não foi significativa pelo teste de hipótese de McNemar (p=0,289). Apenas 10 (4,4%) amostras foram positivas em ambos os métodos ( Tabela 1 ).

**Tabela 1:** Descrição geral dos resultados dos exames para anticorpos anticitoplasma de neutrófilos

Resultados	n (%)	Mediana (mínimo-máximo)
IFI		
Negativo	211 (93,0)	
Positivo	16 (7,0)	
Padrão dos positivos		
aANCA	3 (1,3)	
cANCA	8 (3,5)	
pANCA	5 (2,2)	
Mieloperoxidase (unidade)		2,34 (0,97-46,20)
Categorias de positividade		
Até 20	222 (97,8)	
21-30	3 (1,3)	
>30	2 (0,9)	
Proteinase 3 (unidade)		2,40 (0,69-116,00)
Categorias de positividade		
Até 20	218 (96,0)	
21-30	2 (0,9)	
>30	7 (3,1)	
ELISA		
Negativo	215 (94,7)	
Positivo	12 (5,3)	
Categorias de positividade		
MPO e PR3 até 20	215 (94,7)	
MPO ou PR3 21-30	3 (1,3)	
MPO ou PR3 >30	9 (4,0)	

IFI: imunofluorescência indireta; aANCA: padrão atípico dos anticorpos anticitoplasma de neutrófilo; cANCA: padrão citoplasmático dos anticorpos anticitoplasma de neutrófilo; pANCA: padrão perinuclear dos anticorpos anticitoplasma de neutrófilo; ELISA: ensaio imunoenzimático; MPO: mieloperoxidase; PR3: proteinase 3.

Dos 16 casos positivos pela IFI, 8 foram classificados como padrão cANCA ( Figura 1 ), dos quais 6 foram positivos para anti-PR3 e 2 foram negativos nos testes de ELISA; 5 casos foram classificados como padrão pANCA ( Figura 2 ), dos quais 3 foram positivos para anti-MPO, 1 positivo para anti-PR3 e 1 foi negativo em ambos os testes de ELISA; três foram classificados como padrão aANCA ( Figura 3 ), também negativos para os testes de ELISA.

**Figura 1:** Padrão citoplasmático observado à imunofluorescência indireta em neutrófilos humanos fixados com etanol. Observa-se fluorescência verde-maçã em todo o citoplasma dos neutrófilos segmentados, com núcleos negativos

**Figura 2:** Padrão perinuclear observado à imunofluorescência indireta em neutrófilos humanos fixados com etanol. Observa-se fluorescência verde-maçã ao redor e sobre os núcleos dos neutrófilos segmentados, com o restante do citoplasma negativo

**Figura 3:** Padrão atípico observado à imunofluorescência indireta em neutrófilos humanos fixados com etanol. Observa-se fluorescência verde-maçã com características que não a definem como um dos dois padrões clássicos de fluorescência (citoplasmático ou perinuclear)

Adicionalmente, dois casos negativos na IFI foram positivos para ELISA anti-PR3, um com valor entre 21 e 30 unidades e um com valor acima de 30 unidades ( Tabela 2 ).

**Tabela 2:** Resultados dos exames de ensaio imunoenzimático para anticorpos anticitoplasma de neutrófilos

Ensaio imunoenzimático	Imunofluorescência indireta				

	Negativo	Positivo	aANCA	cANCA	pANCA
Negativo	209 (99,1)	6 (37,5)	3 (100,0)	2 (25,0)	1 (20,0)
Positivo	2 (0,9)	10 (62,5)	0 (0,0)	6 (75,0)	4 (80,0)
Categorias de positividade					
MPO e PR3 até 20	209 (99,1)	6 (37,5)	3 (100,0)	2 (25,0)	1 (20,0)
MPO ou PR3 21-30	1 (0,5)	2 (12,5)	0 (0,0)	1 (12,5)	1 (20,0)
MPO ou PR3 >30	1 (0,5)	8 (50,0)	0 (0,0)	5 (62,5)	3 (60,0)
Mieloperoxidase (unidade)					
Até 20	211 (100,0)	11 (68,8)	3 (100,0)	6 (75,0)	2 (40,0)
21-30	0 (0,0)	3 (18,8)	0 (0,0)	2 (25,0)	1 (20,0)
>30	0 (0,0)	2 (12,5)	0 (0,0)	0 (0,0)	2 (40,0)
Protease de serina 3 (unidade)					
Até 20	209 (99,1)	9 (56,2)	3 (100,0)	2 (25,0)	4 (80,0)
21-30	1 (0,5)	1 (6,2)	0 (0,0)	1 (12,5)	0 (0,0)
>30	1 (0,5)	6 (37,5)	0 (0,0)	5 (62,5)	1 (20,0)

Resultados expressos como n (%). aANCA: padrão atípico dos anticorpos anticitoplasma de neutrófilos; cANCA: padrão citoplasmático dos anticorpos anticitoplasma de neutrófilos; pANCA: padrão perinuclear dos anticorpos anticitoplasma de neutrófilos; MPO: mieloperoxidase; PR3: proteinase 3.

Os dois métodos foram concordantes em 10 amostras positivas e em 209 negativas, resultando em concordância de 96,5% e coeficiente Kappa de 0,70 (IC95%: 0,50-0,90; p<0,001). Esse valor indica concordância substancial, mas não perfeita.

A tabela 3 apresenta as medidas diagnósticas do teste ELISA para ANCA, tendo a IFI como padrão-ouro, em que se nota que sua sensibilidade foi de 0,62 e especificidade de 0,99, mostrando-se um teste mais específico do que sensível, com acurácia diagnóstica em 96% dos casos.

**Tabela 3:** Medidas diagnósticas do teste de ensaio imunoenzimático para anticorpos anticitoplasma de neutrófilos, de acordo com imunofluorescência indireta

Medidas diagnósticas	Estimativa
Sensibilidade	0,62 (0,35-0,85)
Especificidade	0,99 (0,97-1,00)
Valor preditivo positivo	0,83 (0,52-0,98)
Valor preditivo negativo	0,97 (0,94-0,99)
Acurácia diagnóstica	0,96 (0,93-0,98)

As medidas foram avaliadas a partir de tabelas de contingência.

## DISCUSSÃO

Resultados negativos ao ELISA foram encontrados em amostras positivas à IFI com todos os padrões de fluorescência (cANCA, pANCA e aANCA), mas mais frequentemente nos padrões aANCA. Este achado difere do encontrado por Damoiseaux et al., que sugeriram não haver vantagem no uso da IFI, como triagem ou em associação ao ELISA, para a pesquisa de ANCA em pacientes com suspeição diagnóstica de vasculites.^12^

Harris et al., demonstraram que, em casos de vasculite necrotizante sistêmica ativa confirmada por biópsia, o método de ELISA teve sensibilidade semelhante (85% *versus* 88%; p=0,056), porém maior especificidade (97% *versus* 90%; p=0,0006) e valor preditivo positivo (73% *versus* 50%; p=0,0013) do que a IFI para a detecção de ANCA. Os autores também mostraram que a combinação de imunofluorescência e ELISA levou a um valor preditivo positivo menor em comparação à realização apenas do ELISA.^15^ Nossos resultados, porém, sugerem que a realização da IFI em todos os casos encaminhados para a pesquisa de ANCA aumentaria a sensibilidade dos testes, pois classificaria como positivos pacientes que, sem o uso da IFI, seriam considerados negativos.

Por outro lado, a ocorrência de duas amostras negativas à IFI, com positividade para anti-PR3, um autoanticorpo de alta relevância clínica para o diagnóstico de GPA, sugere que a pesquisa de anti-PR3 por ELISA previamente ou simultaneamente à realização da IFI pode agregar poder diagnóstico ao teste, conforme apresentado em estudos anteriores.^4 , 6^

Esses dados revelam a necessidade da revisão do algoritmo diagnóstico utilizado atualmente, corroborando achados de estudos prévios.^13 , 14^

Nosso estudo, entretanto, possui limitações, como a falta de avaliação da correlação entre os resultados dos testes laboratoriais e o diagnóstico clínico dos indivíduos testados. Pretendemos avaliar esta correlação em estudos futuros. Além disso, a IFI foi analisada como padrão-ouro, não sendo possível estimar a sensibilidade e a especificidade desse método isoladamente.

Em nossa casuística, o uso combinado de ELISA e IFI simultaneamente seria a estratégia diagnóstica mais adequada, conferindo maior sensibilidade ao teste de ANCA e incluindo como positivos todos os pacientes com autoanticorpos de especificidades antigênicas relevantes para o diagnóstico diferencial de vasculites sistêmicas, particularmente aqueles com positividade para anti-PR3.

## CONCLUSÃO

Os resultados demonstraram que os métodos de ELISA antimieloperoxidase e antiproteinase 3 utilizados em conjunto tiveram alta especificidade, mas sensibilidade inferior.

É possível concluir que a realização da imunofluorescência indireta aumentou a sensibilidade do teste de ANCA em relação ao uso isolado do ELISA, ao mesmo tempo em que a pesquisa de antiproteinase 3 por ELISA também agregou poder de sensibilidade para amostras com esta especificidade antigênica. Com base nestes resultados, é possível sugerir que o uso combinado de imunofluorescência indireta e ELISA simultaneamente seria a estratégia diagnóstica mais adequada para a população estudada.
